# Casein Kinase 1 Phosphomimetic Mutations Negatively Impact Connexin-43 Gap Junctions in Human Pluripotent Stem Cell-Derived Cardiomyocytes

**DOI:** 10.3390/biom14010061

**Published:** 2024-01-02

**Authors:** Rasha Al-attar, Joseph Jargstorf, Rocco Romagnuolo, Mariam Jouni, Faisal J. Alibhai, Paul D. Lampe, Joell L. Solan, Michael A. Laflamme

**Affiliations:** 1 McEwen Stem Cell Institute, University Health Network, Toronto, ON M5G 1L7, Canada; ral-attar@mgh.harvard.edu (R.A.-a.); joseph.jargstorf@gmail.com (J.J.); rromagnuolo@bluerocktx.com (R.R.); mariam.m.jouni@gmail.com (M.J.); faisal.alibhai@uhn.ca (F.J.A.); 2 Translational Research Program, Public Health Sciences and Human Biology Divisions, Fred Hutchinson Cancer Research Center, Seattle, WA 98109, USA; plampe@fredhutch.org (P.D.L.); jsolan@fredhutch.org (J.L.S.); 3 Peter Munk Cardiac Centre, University Health Network, Toronto, ON M5G 1L7, Canada; 4 Department of Laboratory Medicine and Pathobiology, University of Toronto, Toronto, ON M5G 1L7, Canada

**Keywords:** connexin 43, gap junctions, human pluripotent stem cell-derived cardiomyocytes, phosphorylation

## Abstract

The transplantation of human pluripotent stem cell-derived cardiomyocytes (hPSC-CMs) has shown promise in preclinical models of myocardial infarction, but graft myocardium exhibits incomplete host–graft electromechanical integration and a propensity for pro-arrhythmic behavior. Perhaps contributing to this situation, hPSC-CM grafts show low expression of connexin 43 (Cx43), the major gap junction (GJ) protein, in ventricular myocardia. We hypothesized that Cx43 expression and function could be rescued by engineering Cx43 in hPSC-CMs with a series of phosphatase-resistant mutations at three casein kinase 1 phosphorylation sites (Cx43-S3E) that have been previously reported to stabilize Cx43 GJs and reduce arrhythmias in transgenic mice. However, contrary to our predictions, transgenic Cx43-S3E hPSC-CMs exhibited reduced Cx43 expression relative to wild-type cells, both at baseline and following ischemic challenge. Cx43-S3E hPSC-CMs showed correspondingly slower conduction velocities, increased automaticity, and differential expression of other connexin isoforms and various genes involved in cardiac excitation–contraction coupling. Cx43-S3E hPSC-CMs also had phosphorylation marks associated with Cx43 GJ internalization, a finding that may account for their impaired GJ localization. Taken collectively, our data indicate that the Cx43-S3E mutation behaves differently in hPSC-CMs than in adult mouse ventricular myocytes and that multiple biological factors likely need to be addressed synchronously to ensure proper Cx43 expression, localization, and function.

## 1. Introduction

The adult human heart has very limited regenerative capacity, so muscle lost during a myocardial infarction (MI) is replaced by scar tissue, often initiating progressive heart failure [1]. Currently available therapies for post-MI heart failure are largely limited to easing symptoms and/or slowing disease progression, and organ transplantation remains the only means of replacing lost myocardium. This situation and the limited supply of suitable donor hearts have prompted considerable recent interest in harnessing human pluripotent stem cells (hPSCs) as an essentially inexhaustible source of replacement cardiomyocytes. Encouraging results have been reported in preclinical studies, and the intra-cardiac transplantation of hPSC-derived cardiomyocytes (hPSC-CMs) has been shown to mediate the partial remuscularization of the infarct scar and beneficial effects on left ventricular contractile function in both small and large animal MI models [2,3,4,5,6,7,8,9,10,11]. Importantly, the hPSC-CM graft tissue formed in these models is capable of electromechanical integration and synchronous 1:1 coupling with host myocardia [2,3,4,5,6,7,8]. On the other hand, host–graft integration is far from perfect, and our group has performed optical mapping of infarcted hearts with hPSC-CM grafts expressing genetically encoded calcium- and voltage-sensitive fluorescent reporters and observed 1:1 coupling in only a subset of recipient hearts and, usually then, only in a portion of the visible graft tissue [3,6,7,8]. Moreover, when conduction velocity (CV) in graft tissue has been measured in these same mapping studies, it has proven slow, typically only one-fifth to one-tenth of the CV in adult ventricular myocardium [4,5]. Likely accounting—at least in part—for the poor coupling and slow propagation in hPSC-CM graft tissue, all published transplantation studies have reported relatively low-level expressions of the gap junction (GJ) protein connexin 43 (Cx43) in engrafted hPSC-CMs [2,3,4,5,6,7]. Using immunohistochemistry, Cx43 expression in engrafted hPSC-CMs is usually found at vanishingly low levels relative to host cardiomyocytes and is isotropically distributed in the sarcolemma rather than enriched in intercalated discs.

GJs are transmembrane complexes of connexin proteins that form intercellular channels between adjacent cells and allow the direct diffusion of ions and small (<1 kDa) molecules [12]. During GJ assembly, six connexins oligomerize to form a hemichannel (or connexon), which is then trafficked to the plasma membrane. When two hemichannels from adjacent cells dock at a point of close cell apposition, a GJ channel is formed that spans two plasma membranes and the narrow intercellular “gap”. Tens or thousands of such channels typically cluster to form GJ plaques that can be recognized at the ultrastructural or light microscopic level. Cx43, encoded for by the *GJA1* gene, is the most abundant connexin isoform in ventricular cardiomyocytes, in which it plays a critical role in action potential (AP) propagation and has non-canonical functions, including the regulation of cardiac ion channel trafficking, intercellular adhesion, and cell cycle activity [12,13,14]. Given these important functions, it is perhaps unsurprising that Cx43 GJs are tightly regulated at the transcriptional [15,16,17,18], post-transcriptional [19,20,21,22,23], and post-translational [24,25,26,27,28,29,30,31,32,33,34,35,36,37,38] levels to ensure appropriate expression, trafficking, and function.

Phosphorylation events play a particularly important role in Cx43 GJ assembly, gating, and degradation, and many of the 21 serine (S) residues in the carboxy terminus of Cx43 have been reported to be the phosphorylation targets of various kinases, including protein kinase B (PKB or Akt), protein kinase C (PKC), mitogen-activated protein kinase (MAPK), and casein kinase 1 (CK1) [28]. Several studies have shown that acute stressors including ischemia promote the internalization of Cx43 GJs by modifying the phosphorylation status of Cx43 [38,39,40], reducing cell–cell communication. In adult ventricular cardiomyocytes, ischemia mediates a decrease in both total and CK1-phosphorylated forms of Cx43, resulting in decreased sarcolemmal Cx43 and impaired electrical coupling between neighboring myocytes [40]. In an intriguing study, Remo and colleagues focused on the role of CK1 phosphorylation in cardiac Cx43 GJ remodeling and created transgenic mice in which three Cx43 CK1 phosphorylation sites (S325/328/330) were replaced by phosphomimetic glutamic acids (hereafter abbreviated as Cx43-S3E) [41]. Interestingly, these authors found that the transgenic mice with the phosphatase-resistant Cx43-S3E mutation were resistant to ischemia-induced Cx43 GJ remodeling and had greatly reduced susceptibility to induced ventricular tachyarrhythmias [41]. Mice with the Cx43-S3E mutation also showed attenuated Cx43 GJ remodeling and arrhythmia vulnerability when crossed with the mdx model of Duchenne muscular dystrophy [42].

Inspired by this work in mouse models, we hypothesized that engineering hPSC-CMs with the corresponding Cx43-S3E mutation would result in larger, more ischemia-resistant Cx43 GJs and more stable Cx43 expression in hPSC-CM graft tissue. To test this, we used CRISPR/Cas9-mediated gene editing to modify the endogenous Cx43 locus and created transgenic hPSC-CMs bearing the Cx43-S3E mutation. The phenotype of Cx43-S3E and wild-type (WT) hPSC-CMs were then compared using parameters, including Cx43 expression and subcellular localization (immunocytochemistry), electrophysiological and calcium handling properties (optical mapping), and cardiac gene expression (qRT-PCR). In contrast to our expectations, we found that Cx43-S3E hPSC-CMs exhibited reduced membranous Cx43, worsened electrophysiological function, and interesting compensatory changes in multiple essential cardiac genes.

## 2. Materials and Methods

### 2.1. Generation of Transgenic Cx43-S3E hPSCs

**Figure 1:**
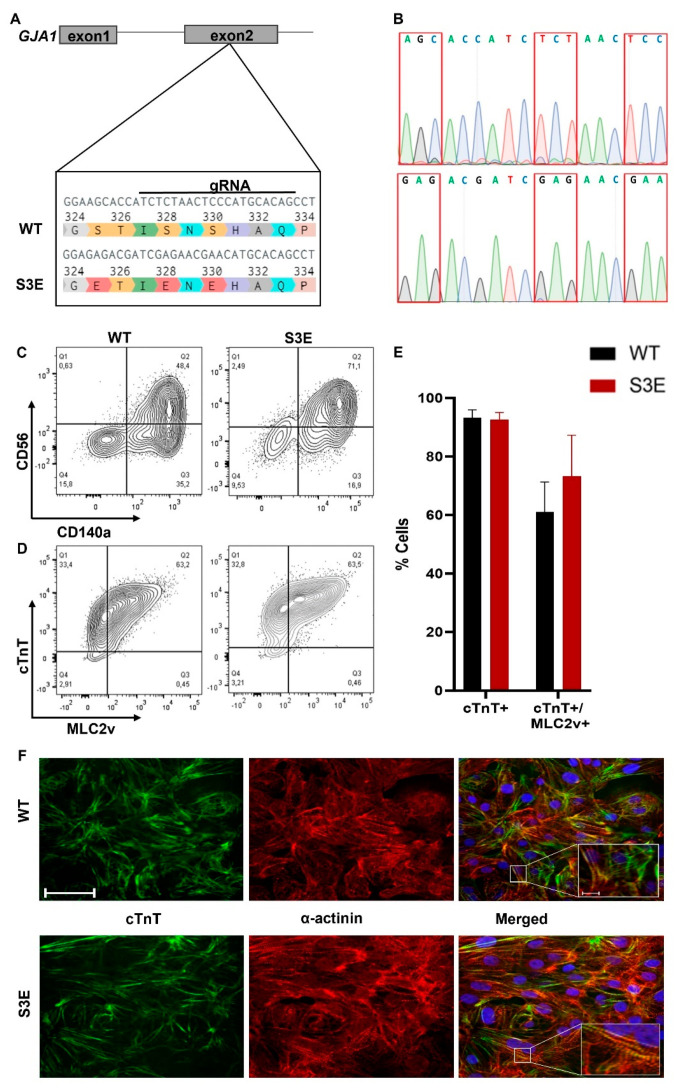
Generation and cardiac differentiation of transgenic Cx43-S3E hPSCs. (**A**) Schematic of CRISPR/Cas9 genome editing strategy used to establish the stable Cx43-S3E hPSC line. In brief, hPSCs were co-transfected with a plasmid encoding for the Cas9 nuclease and a gRNA targeting a PAM motif between S325 and S328 in the second exon of the *GJA1* gene and a single-stranded donor template including the three indicated glutamic acid substitutions (S to E). (**B**) Sanger sequencing of WT (upper) and Cx43-S3E (lower) hPSCs, confirming successful targeting in the latter. (**C**,**D**) Representative flow cytograms demonstrating comparably high expression of mesodermal markers CD56 and CD140a (**C**) and the pan-cardiac marker cTnT and ventricular marker MLC2v (**D**) from differentiating WT and Cx43-S3E hPSC cultures on days 3 and 20 post-induction, respectively. (**E**) % of cells expressing cTnT, and cTnT and MLC2v in differentiated WT vs. Cx43-S3E cultures. (**F**) Confocal photomicrographs of representative WT and Cx43-S3E hPSC-CMs dual-immunostained for cTnT (green) and α-actinin (red). Scale bar = 50 µm.

We used CRISPR/Cas9-mediated gene editing [43] to introduce the S3E mutations into the endogenous Cx43 gene (*GJA1*) in the ESI-17 human embryonic stem cell line (Biotime, Alameda, CA, USA) [44,45]. In brief, a custom gRNA targeting a protospacer adjacent motif (PAM) between serine-325 and serine-328 of the *GJA1* gene was cloned into a single plasmid that also encoded for the Cas9 nuclease and a green fluorescent protein (GFP) reporter (Addgene plasmid #48138, Watertown, MA, USA) [43]. A single-stranded donor nucleotide sequence was designed and synthesized with the following characteristics: glutamic acid substitutions at the three CK1 phosphorylation sites (S325E, S328E, and S330E), a silent mutation to introduce a unique BsiEI restriction digest site, and a second silent mutation to mutate the PAM sequence (Figure 1A). ESI-17 hPSCs were co-transfected (XtremeGENE 9, Millipore Sigma, Burlington, MA, USA) with both constructs, and the resulting green fluorescent clones were sorted and expanded. Proper targeting of the Cx43 locus was confirmed using PCR amplification of the genomic DNA region encompassing the mutations of interest, followed by restriction digest with the BsiEI restriction enzyme and gel electrophoresis. Successfully targeted homozygous clones were validated with Sanger sequencing (Centre for Applied Genomics, The Hospital for Sick Children, Toronto, ON, Canada), karyotyped (Medical Genetics Laboratories, Cambridge University Hospitals NHS, Cambridge, UK), and expanded for cardiac differentiation and subsequent phenotyping.

### 2.2. Cardiac Differentiation of hPSCs

Homozygously targeted Cx43-S3E and WT hPSCs were expanded in the undifferentiated state using mTeSR1 medium (Stem Cell Technologies, Vancouver, BC, Canada). We applied a slightly modified version of a previously reported growth factor-based cardiac differentiation protocol to induce cardiogenesis [5,46]. In brief, undifferentiated hPSCs were enzymatically dispersed using TrypLE (Thermo Fisher Scientific, Waltham, MA, USA) and then transferred to suspension culture at a density of 1 × 10^6^ cells/mL on an orbital shaker (75 rpm). During this aggregation step, cells were cultured in a basal medium consisting of StemPro 34 medium (Thermo Fisher Scientific) with L-glutamine (2 mM, Fisher), L-ascorbic acid (50 µg/mL, Sigma, St. Louis, MO, USA), monothioglycerol (50 µg/mL, Sigma), and transferrin (150 µg/mL, Roche, Basel, Switzerland) supplemented with bone morphogenetic protein-4 (BMP4, 1 ng/mL, R&D Systems, Minneapolis, MN, USA) and Rho-associated protein kinase (ROCK) inhibitor Y-27632 (RI; 10 µM, StemCell Technologies) in a low oxygen environment (5% O_2_). After 24 h, the resultant aggregates were transferred to an induction medium composed of the preceding basal medium but supplemented here with BMP4 (10 ng/mL), activin A (6 ng/mL, R&D Systems), and basic fibroblast growth factor (bFGF, 5 ng/mL, Peprotech, Cranbury, NJ, USA). On day 3 of differentiation, aggregates were changed to basal medium supplemented with Wnt inhibitor IWP2 (2 µM, Tocris, Bristol, UK) and vascular endothelial growth factor (VEGF, 10 ng/mL, Peprotech) and cultured for an additional 3 days. On day 6 of differentiation, aggregates were harvested, dissociated to single cells with the TrypLE enzyme, and replated at 1.2 × 10^5^ cells/cm^2^ in 6-well tissue culture plates coated with growth factor-reduced Matrigel (Corning, Corning, NY, USA) in basal medium supplemented with VEGF (5 ng/mL) and RI (10 uM). One day later, differentiating cultures were transferred and maintained in basal medium with VEGF (5 ng/mL) but no RI until spontaneous beating was initiated (~day 10–12). Thereafter, cells were switched to a maintenance medium consisting of RPMI medium supplemented with B-27 (with insulin) and 2 mM of L-glutamine until day 20. hPSC-CM cultures were then dissociated using 0.125% (*w*/*v*) trypsin and employed in phenotyping experiments.

### 2.3. Flow Cytometry for Mesodermal and Cardiac Markers

Differentiating cultures were routinely assessed using an LSRII/Fortessa flow cytometer system (BD PharMingen, Franklin Lakes, NJ, USA) and primary antibodies against mesodermal markers CD56 and CD140a and cardiomyocyte markers cardiac troponin T (cTnT) and myosin light chain 2v (MLC2v) as previously reported [47]. Titers for each primary antibody are listed in Supplemental Table S1. All experiments were performed using hPSC-CM populations with at least 90% cardiomyocyte purity as determined using flow cytometry for the pan-cardiomyocyte marker cTnT.

### 2.4. Immunofluorescence Staining

In order to evaluate the expression and subcellular localization of targets of interest using immunofluorescence microscopy, undifferentiated hPSCs or hPSC-CMs were seeded onto glass coverslips and cultured for at least 72 h prior to fixation with 4% (*w*/*v*) paraformaldehyde for 10 min at room temperature. Fixed cultures were washed with PBS, blocked with 10% normal goat serum (Vector Labs, Newark, CA, USA supplemented with 0.1% Triton X-100), and immunostained with the primary antibodies against various markers of interest overnight at 4 °C. Undifferentiated hPSC cultures were stained with primary antibodies against pluripotency markers including SSEA4, SOX2, OCT-4A, NANOG, all purchased from Cell Signaling Technology (Danvers, MA, USA). hPSC-CMs were stained with primary antibodies against Cx43, cTnT, and sarcomeric α-actinin. Titers and catalogue numbers for each primary antibody are listed in Supplemental Table S1. Cells were washed and stained with appropriate fluorescently tagged, species-specific secondary antibodies (Alexa Fluor 488-, 594-, or 647-conjugated, ThermoFisher Scientific, Waltham, MA, USA) for 1.5 h at room temperature next. After nuclear counterstaining (Hoechst, Sigma-Aldrich, St. Louis, MO, USA), mounting (VectaShield, VectorLabs), and coverslipping, immunostained monolayers were imaged using a Nikon A1R confocal microscope (Advanced Optical Microscopy Facility, University Health network, Toronto, ON, Canada). All quantitative immunofluorescence data reflect 8–10 randomly selected fields from at least 3 biological replicates per condition. Parameters including total, peri-nuclear, and membranous Cx43 expression were manually evaluated using ImageJ software (version v1.54h).

### 2.5. Fluorescent Voltage and Calcium Imaging

Optical mapping of fluorescent voltage and calcium signals was performed on hPSC-CM monolayers loaded with either the voltage-sensitive indicator Fluovolt (Thermo Fisher Scientific) or the calcium-sensitive indicator Fluo-4 AM (Thermo Fisher Scientific). For these experiments, compact monolayers of hPSC-CMs were formed by replating onto growth factor-reduced Matrigel-coated coverslips at 1.2 × 10^5^ cells/cm^2^ and cultured for at least 72 h prior to imaging in RPMI-B27 medium. Where indicated, hPSC-CM cultures were exposed immediately before mapping experiments using an in vitro ischemic challenge consisting of a 120 min exposure to hypoxia (1% O_2_) in an ischemia buffer consisting of (in mM) 118 NaCl, 1.0 NaH_2_PO_4_•H_2_O, 2.5 CaCl_2_, 1.2 MgCl_2_, 0.5 Na_2_EDTA•2H_2_O, 20 Na-lactate, 16 KCl, 30 MES hydrate [2-(N-morpholino)ethanesulfonic acid hydrate, 4-morpholineethanesulfonic acid)] and the pH adjusted to 6.3. We chose a 2 h duration for this ischemic challenge because Cx43 proteins reportedly have a half-life of approximately 90–120 min [48,49]. Shortly before imaging, cultures were loaded with either Fluovolt (5 µM) or Fluo-4 AM (10 µM) for 30 min; in the case of ischemic conditions, dye loading commenced at the 90 min time point during ischemia. During imaging, cultures were maintained at 37 °C in a modified Tyrode buffer consisting of (in mM) 140 NaCl, 1.0 MgCl_2_, 0.33 NaH_2_•PO_4_H_2_O, 5.4 KCl, 5.0 D-Glucose, 10 HEPES, 1.8 CaCl_2_•H_2_O adjusted to pH 7.4.

Fluorescent voltage and calcium signals were acquired using a previously described [4,5] optical mapping rig comprising a fluorescence microscope (Olympus MVX10 MacroZoom, 0.63X objective with 13 × 13 mm field of view, 100 µm per pixel) outfitted with a high-speed, high-sensitivity 128 × 128 pixel EMCCD camera (Evolve128, Photometrics, Tucson, AZ, USA). Excitation was provided with an LED spotlight (Mightex PLS-0470-030-150S with ET470/40X Chroma filter, Toronto, ON, Canada), and emitted signals were bandpass-filtered to 500–530 nm and acquired at 500 frames per second. A previously established custom MatLab (MathWorks, Natick, MA, USA) script was used to analyze the data and generate activation maps [4,5,50]. The entirety of the monolayer was used for analysis and at least 5 regions of interest per monolayer were randomly selected for calculating the various parameters.

### 2.6. Western Blot

Whole-cell protein lysates were obtained using RIPA lysis buffer (Abcam, Waltham, MA, USA) as per vendor instructions. For each condition examined, 25 µg of protein were loaded and separated using a 15% Tris-glycine gel, then transferred to polyvinylidene difluoride membranes and processed as previously described [51]. Immunoblots were probed with the following antibodies at 1:1000 dilution: Cx43 (total) and p-Cx43 (S279/282) [52].

### 2.7. Gene Expression

Total RNA was extracted from day 20 hPSC-CMs using TRIzol (BioShop, Burlington, ON, Canada), and RNA was reverse transcribed using iScript Reverse Transcription Supermix (Bio-Rad, Hercules, CA, USA) as per vendor instructions. Quantitative real-time polymerase chain reaction (qRT-PCR) was performed as previously described [51]. The DNA damage binding protein 1 (DDB1) gene showed stable expression between WT and the S3E mutants and was used as a reference gene. The list of primers used can be found in Supplemental Table S2.

### 2.8. Statistical Analysis

All data were analyzed in a blind manner with breaking of the identifier code only after analysis was completed. All plots depict mean ± SEM from at least *n* = 3 independent differentiations unless otherwise stated. Statistical analyses were performed using GraphPad Prism 9 software (Boston, MA, USA). Statistical comparisons were calculated either using a two-way ANOVA followed by a Tukey post hoc test or an unpaired Student t-test. The threshold for statistical significance was set at level *p* < 0.05.

## 3. Results

### 3.1. Generation and Characterization of Cx43-S3E hPSCs

We used CRISPR-Cas9-mediated gene editing to create transgenic hPSCs in which the three CK1 phosphorylation sites were replaced by glutamic acids (S325E, S328E, and S330E) at both alleles of the Cx43 gene (*GJA1*) (Figure 1A,B). We selected and expanded a Cx43-S3E hPSC clone that showed a normal karyotype (Figure S1) and unaltered expression of the pluripotency markers NANOG, Sox2, OCT4, and SSEA-4 (Supplemental Figure S2).

### 3.2. Cardiac Differentiation of Cx43-S3E hPSCs

We successfully differentiated both transgenic Cx43-S3E and WT hPSCs into cardiomyocytes using a previously reported growth factor-based protocol [5]. After 3 days under differentiating conditions, both Cx43-S3E and WT cultures included comparable fractions (~50–70%) of CD140a and CD56 double-positive cells (Figure 1C), indicating efficient mesoderm induction and cardiac progenitor specification. Spontaneous beating was observed in both Cx43-S3E and WT cultures after 10–12 days of differentiation. After 20 days under differentiating conditions, both Cx43-S3E and WT cultures consisted of highly pure populations of ventricular myocytes as demonstrated by >90% co-expression of the pan-cardiac marker cTnT and ventricular-specific marker MLC2v (Figure 1D). There was no significant difference in the fraction of cTnT^+^ cardiomyocytes or cTnT^+^/MLC2v^+^ ventricular myocytes between the two conditions (Figure 1E). Cardiomyocytes from both lines had comparable morphologies and sarcomeric structures (Figure 1F, Supplemental Video S1).

### 3.3. Cx43 Expression and Localization in Cx43-S3E versus WT hPSC-CMs

**Figure 2:**
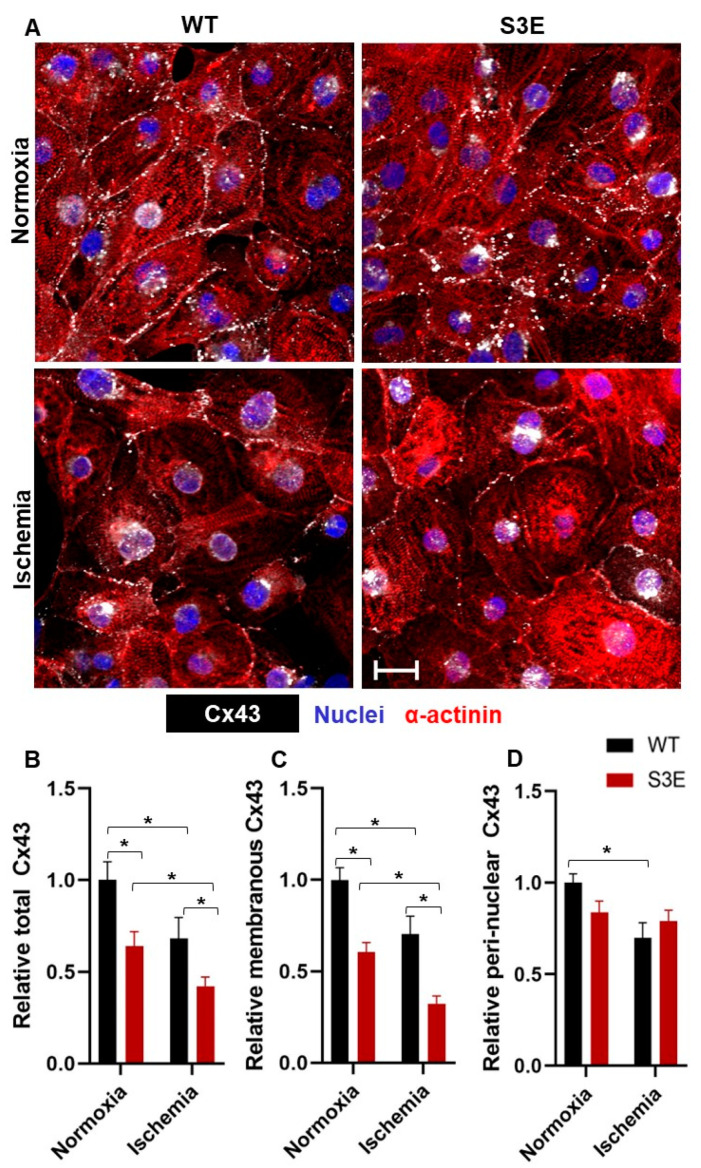
Cx43 expression and subcellular location are impaired in Cx43-S3E hPSC-CMs. (**A**) Confocal photomicrographs of representative WT and Cx43-S3E hPSC-CMs cultures under baseline normoxia and ischemic conditions dual-immunostained for Cx43 (white) and α-actinin (red). Scale bar = 10 µm (**B**–**D**). Quantitation of total (**B**), membranous (**C**), and peri-nuclear (**D**) Cx43 immunoreactivity in each of the four experimental conditions. All data are presented as mean ± SEM from *n* = 3–4 biological replicates; * *p* < 0.05 as analyzed using two-way ANOVA with Tukey’s HSD post hoc test.

We next used quantitative confocal microscopy to compare the expression and subcellular localization of Cx43 in Cx43-S3E versus WT hPSC-CMs, both at baseline and following in vitro ischemic challenge (Figure 2A). Interestingly, under baseline (i.e., normoxic control) conditions, total Cx43 expression was significantly reduced in Cx43-S3E hPSC-CMs to only 0.64 ± 0.07-fold of that in WT hPSC-CMs (Figure 2B). Moreover, although we had hypothesized that Cx43 would show ischemia resistance in the transgenic cardiomyocytes, both WT and Cx43-S3E hPSC-CMs showed a significant reduction in total Cx43 expression relative to their baseline counterparts following ischemia (to 0.68 ± 0.1 and 0.42 ± 0.05-fold of baseline expression, respectively). This same pattern held when analyzing membranous Cx43 immunoreactivity: Cx43-S3E hPSC-CMs showed 0.61 ± 0.05-fold less membranous Cx43 than WT CMs at baseline, and they showed an even greater reduction in membranous Cx43 following ischemia (with membranous Cx43 in Cx43-S3E and WT hPSC-CMs declining to 0.26 ± 0.04 and 0.70 ± 0.09-fold of baseline WT controls, respectively) (Figure 2C). Lastly, we evaluated the peri-nuclear Cx43 localization under these same four conditions. Interestingly, while there was no significant difference in peri-nuclear Cx43 between WT and S3E mutants at baseline or between S3E mutants at normoxia or ischemia, WT hPSC-CMs did show a reduction in peri-nuclear Cx43 following ischemia (Figure 2D).

### 3.4. Electrophysiology and Intracellular Calcium Handling in Cx43-S3E versus WT hPSC-CMs

**Figure 3:**
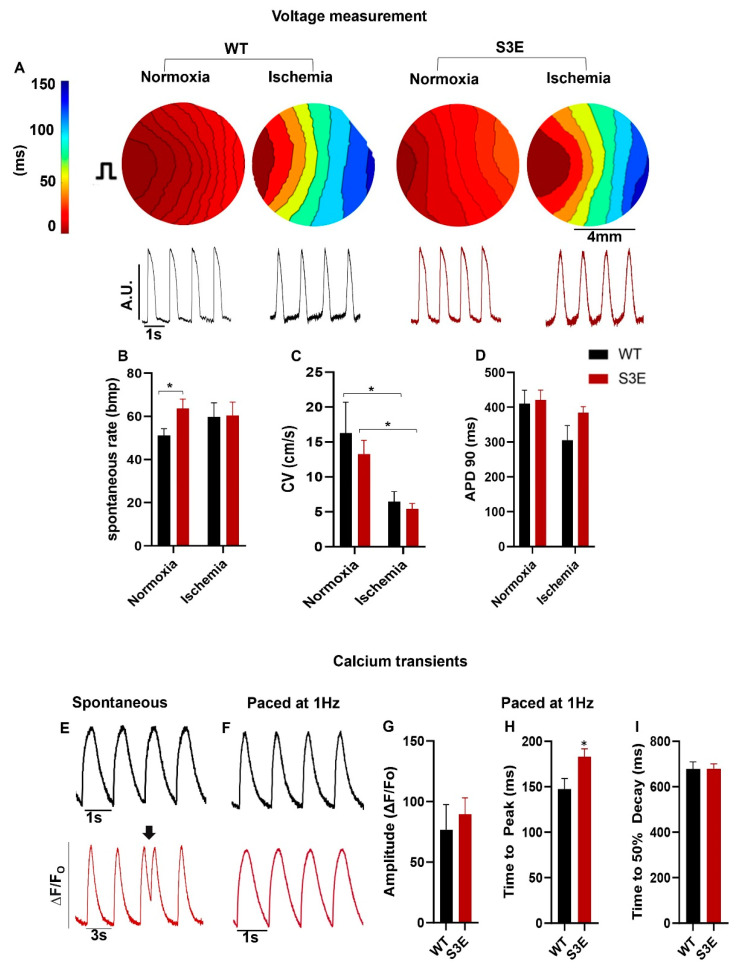
Action potential propagation and [Ca^2+^]_i_ handling are not enhanced in Cx43-S3E hPSC-CMs. (**A**) Upper: Representative activation maps (upper) for WT and Cx43-S3E hPSC-CM monolayers acquired at baseline (normoxia, normal buffer) or after ischemic challenge. Monolayers were paced at 1 Hz with an electrode placed at the 9 o’clock position. Lower: Corresponding optical action potential traces from each condition. (**B**) Spontaneous beating rate in WT and Cx43-S3E hPSC-CM cultures under baseline or ischemic conditions. (**C**,**D**) Other electrophysiological parameters including CV (**C**) and APD90 (**D**) as acquired under paced conditions. (**E**,**F**) Representative [Ca^2+^]_i_ transients from WT (black) and Cx43-S3E (red) hPSC-CMs as acquired under spontaneous (**E**) or paced (**F**) conditions. Arrow in E shows an early [Ca^2+^]_i_ transient (“afterdepolarization”). (**G**–**I**) [Ca^2+^]_i_ transient parameters including amplitude (**G**), time to peak (**H**), and time to 50% decay (**I**) in WT versus Cx43-S3E hPSC-CMs. Data are presented as mean ± SEM from *n* = 4 biological replicates; * *p* < 0.05 as analyzed using two-way ANOVA for voltage comparisons, followed by Tukey’s HSD post hoc test. Significance for [Ca^2+^]_i_ transient parameters was analyzed using Student’s *t*-test (* *p* < 0.05).

To compare the electrophysiological function of Cx43-S3E versus WT hPSC-CMs at baseline and following ischemic challenge, cell monolayers were imaged after loading with the fluorescent voltage indicator FluoVolt (see Figure 3A for representative images). Interestingly, Cx43-S3E hPSC-CMs at baseline showed a significantly faster spontaneous beating rate than their WT counterparts (64.0 ± 4.4 bpm versus 51.0 ± 3.2 bpm), although this difference normalized after ischemia (Figure 3B). We switched to optical mapping of hPSC-CM monolayers under paced rather than spontaneous conditions to assess other parameters, including CV and action potential duration to 90% of repolarization (APD90). Despite the lower levels of total and membranous Cx43 shown by the immunofluorescence in transgenic hPSC-CMs, there was no significant difference in CV between Cx43-S3E and WT hPSC-CMs either at baseline or following ischemic challenge (Figure 3C). On the other hand, the presence of the Cx43-S3E mutation clearly did not rescue propagation during ischemia (with CV before and after ischemia measuring 13.3 ± 1.9 cm/s and 5.4 ± 0.80 cm/s in Cx43-S3E hPSC-CMs versus 16 ± 3.0 cm/s and 6.5 ± 1.4 cm/s in WT cells) (Figure 3C). There was no significant difference in APD90 between Cx43-S3E and WT hPSC-CMs under either normoxic or ischemic conditions (Figure 3D).

Next, given the linkage between Cx43 expression and intracellular calcium [Ca^2+^]_i_ signaling [53], we compared [Ca^2+^]_i_ transient parameters in Cx43-S3E versus WT hPSC-CMs after loading with the fluorescent [Ca^2+^]_i_ indicator Fluo-4 AM. During spontaneous beating, Cx43-S3E hPSC-CMs showed more irregular [Ca^2+^]_i_ cycling with frequent early [Ca^2+^]_i_ transients (Figure 3E, black arrow). However, the latter behavior was not apparent when comparing paced hPSC-CM monolayers, and we found no significant difference in [Ca^2+^]_i_ transient amplitude or time to 50% decay between WT and Cx43-S3E hPSC-CMs with only a modest but significant increase in time to peak in Cx43-S3E hPSC-CMs (183.1 ± 8.6 ms) compared to WT (147.6 ± 11.5 ms) hPSC-CMs. (Figure 3F–I).

### 3.5. Other Transcriptional Changes in Cx43-S3E vs. WT hPSC-CMs

**Figure 4:**
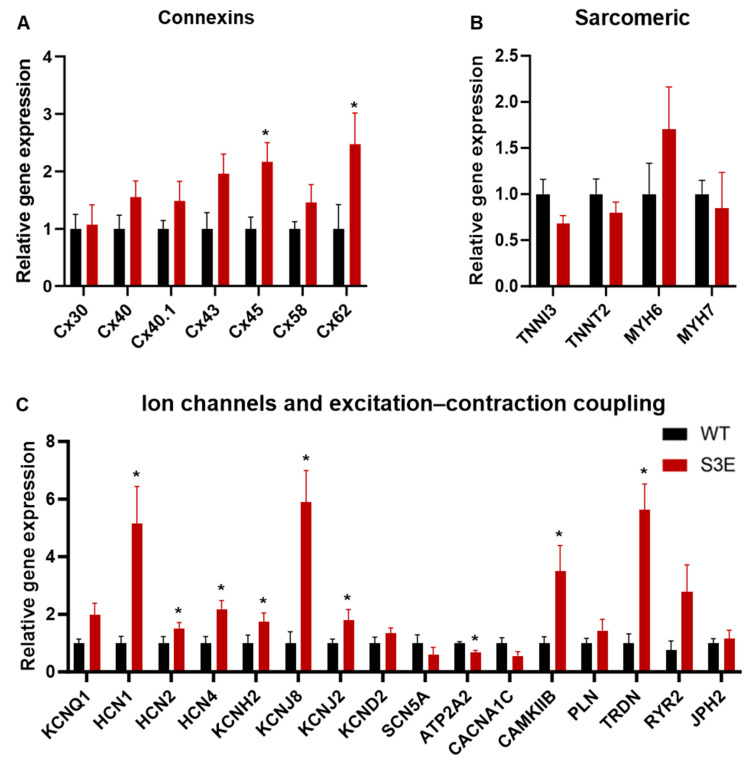
Altered cardiac gene expression in Cx43-S3E versus WT hPSC-CMs. Relative expression of connexin isoforms (**A**), sarcomeric markers (**B**), and genes involved in excitation and/or [Ca^2+^]_i_ handling (**C**) as analyzed using qRT-PCR. Data are presented as mean ± SEM from *n* = 5 biological replicates; * *p* < 0.05 as analyzed using Student’s *t*-test.

Because Cx43 is thought to play a role in regulating gene expression, including the expression of non-GJ-related genes [17,54], we hypothesized that the observed differences in Cx43 expression between Cx43-S3E and WT hPSC-CMs might affect their transcriptional profile. To test this, we performed qRT-PCR with a panel of cardiac markers that included various connexin isoforms (Figure 4A), sarcomeric proteins (Figure 4B), as well as ion channels and excitation–contraction coupling-related targets (Figure 4C). Interestingly, despite the previously mentioned reduction in Cx43 protein, there was a trend toward greater Cx43 transcript in Cx43-S3E vs. WT hPSC-CMs, although this difference did not reach statistical significance. On the other hand, a number of other genes were significantly upregulated between Cx43-S3E versus WT hPSC-CMs including connexin-45 (*GJA7*, 2.2 ± 0.3-fold), connexin-62 (*GJA10*, 2.5 ± 0.5-fold), *HCN4* (2.2 ± 0.3-fold), *KCNH2* (1.8 ± 0.3-fold), *KCNJ8* (5.9 ± 1.1-fold), *KCNJ2* (1.8 ± 0.4-fold), *CAMKIIB* (3.5 ± 0.9-fold), and *TRDN* (5.6 ± 0.9-fold). *ATP2A2* was the sole significantly downregulated gene detected (decreased to 0.7 ± 0.07-fold).

### 3.6. Status of Other Cx43 Phosphorylation Sites in Cx43-S3E vs. WT hPSC-CMs

**Figure 5:**
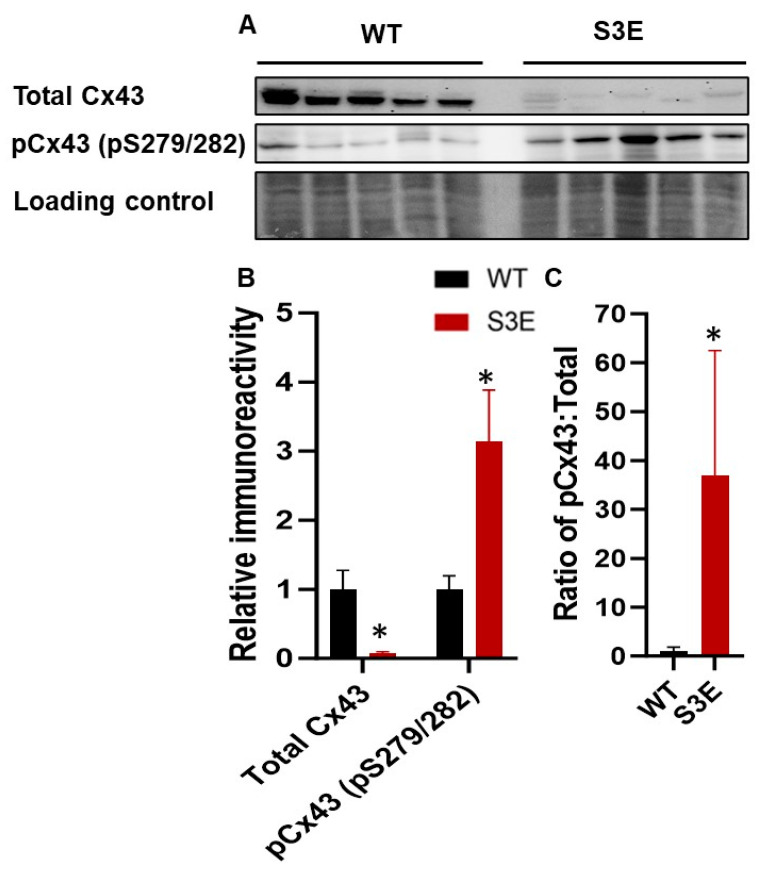
Phosphorylation status of Cx43 at MAPK in Cx43-S3E versus WT hPSC-CMs. (**A**) Immunoblots showing total Cx43, (**B**) phosphorylation on S279/282 in Cx43-S3E versus WT hPSC-CMs, and (**C**) the ratio of pCx43 to total Cx43. Data represented as mean ± SEM from *n* = 5 biological replicates; * *p* < 0.05 as analyzed using Student’s *t*-test.

Because Cx43 trafficking and turnover are regulated by kinases other than CK1 [29] and compensatory phosphorylation events seemed plausible in Cx43-S3E hPSC-CMs, we examined the phosphorylation status of MAPK-regulated residues by immunoblotting with an antibody developed to detect phosphorylation at S279/S282 of Cx43. Several studies have shown that Cx43 phosphorylation by MAPK promotes the internalization of Cx43 GJs and reduces GJ-mediated communication [55], and we found that phosphorylation at two Cx43 sites (S279/282) known to be targeted by MAPK was increased by 3.2 ± 0.74-fold in Cx43-S3E hPSC-CMs relative to WT controls (Figure 5A,B). When normalized to total Cx43, which was decreased in the mutant hPSC-CMs, phosphorylation at these sites was increased 36.9 ± 11.50-fold relative to WT hPSC-CMs (Figure 5C).

## 4. Discussion

The primary objective of the present study was to test the hypothesis that introduction of the Cx43-S3E mutation would improve the stability of membranous Cx43 in gene-edited hPSC-CMs and enhance GJ communication under ischemic conditions. This same mutation had been previously reported to improve Cx43 GJ stability in cardiomyocytes and reduce arrhythmia vulnerability in germline knock-in mice [41]. While our focus here was on comparing in vitro outcomes between Cx43-S3E versus WT hPSC-CMs, we had hoped that the successful demonstration of enhanced Cx43 GJ stability in vitro would provide a strong rationale for an in vivo study to investigate whether Cx43-S3E hPSC-CMs would also exhibit better electromechanical integration and reduced pro-arrhythmic behavior following transplantation in injured hearts [56,57]. Contrary to our hypothesis, Cx43-S3E hPSC-CMs actually had reduced total and membranous Cx43 expression, higher spontaneous beating rates, and a predilection for irregular [Ca^2+^]_i_ cycling. Moreover, rather than exhibiting ischemia resistance, hPSC-CMs engineered with the Cx43-S3E mutation showed an even greater reduction in total and membranous Cx43 expression than their WT counterparts following exposure to in vitro ischemic challenge. Perhaps contributing to their impaired Cx43 expression, the Cx43-S3E gene-edited cardiomyocytes had an altered Cx43 phosphorylation profile at other kinase sites, including increased phosphorylation at two residues (pS279/282) that have been associated with Cx43 GJ disassembly and internalization [27]. Finally, Cx43-S3E hPSC-CMs showed differential expression of other cardiac genes, including upregulated *HCN1* (which encodes for a hyperpolarization-activated cation channel that contributes to spontaneous pacemaker activity) and downregulated *ATP2A2* (which encodes the sarcoplasmic reticulum calcium ATPase that dominates [Ca^2+^]_i_ transient decay) [58]. Both of these changes might be plausibly expected to aggravate automaticity and/or pro-arrhythmic behavior [59]. Additionally, the extended duration for intracellular calcium to reach its peak levels suggests potential disruptions in the coordination of excitation–contraction coupling [60]. These irregularities could contribute to automaticity issues and compromised calcium signaling. A delay in this parameter might instigate the formation of reentrant circuits, leading to the untimely initiation of contraction without completing the preceding cycle.

An important question is why introduction of the Cx43-S3E mutation worsened Cx43 trafficking and cellular electrophysiology in hPSC-CMs in the present study while this same mutation improved Cx43 GJ function in transgenic mouse models [41,42]. Remo and co-workers created knock-in mice with the same Cx43-S3E mutation and found that these animals were resistant to pathological cardiac Cx43 GJ remodeling, exhibited more rapid CV following transverse aortic constriction, and had reduced vulnerability to ventricular tachyarrhythmias induced by premature extrastimuli or burst pacing. While there are obvious major differences in terms of both species (human versus mouse) and experimental preparation (in vitro versus intact myocardium) between the present study and this earlier work, we speculate that the cardiomyocyte maturational stage is the key difference. hPSC-CMs have an immature phenotype, akin to cardiomyocytes in the early fetal heart [61]. They are known to differ from their adult counterparts in terms of both cardiomyocyte structure (e.g., with underdeveloped or absent intercalated disks) [61] and baseline kinase activities [62], so it is perhaps unsurprising that phosphorylation events (or phosphomimetic substitutions) in these cells might exert differential effects on Cx43 GJ assembly and trafficking. Indeed, while the post-translational regulation of Cx43 during early cardiac development has not been well studied (and not studied at all in developing human hearts to our knowledge), fetal mouse cardiomyocytes reportedly have reduced levels of both total Cx43 and Cx43 phosphorylated at multiple sites (S325/S328/S330 and S368) relative to adult myocytes [63].

Our immunoblotting analysis of Cx43 phosphorylation at sites away from the S3E mutation suggests one potential mechanism for the impaired expression and subcellular localization of Cx43 in the gene-edited hPSC-CMs. Cx43 is phosphorylated by various kinases, including CK1, MAPK, PKC, Akt, and these post-translational phosphorylation events regulate Cx43 GJ assembly, stability, localization, degradation, and protein–protein interactions [29]. Interestingly, our Cx43-S3E hPSC-CMs showed increased phosphorylation at Cx43 residues S279/S282, which are MAPK phosphorylation sites reported to promote Cx43 GJ disassembly and internalization [55]. If these are compensatory phosphorylation responses that occur in Cx43-S3E hPSC-CMs (but apparently not in adult myocardium in transgenic mice), one would predict them to be deleterious by reducing both Cx43 GJ stability and function. Future transgenic hPSC studies targeting mutations at the S279/282 site will determine if phosphomimetic substitutions here can rescue the functionality of Cx43-SE hPSC-CMs or perhaps even mediate beneficial synergistic effects.

The decreased membranous Cx43 in Cx43-S3E hPSC-CMs may also reflect impaired anterograde trafficking to the cell membrane [64,65,66]. The Cx43 transcript includes several internal initiation sites that give rise to seven different Cx43 isoforms that have been reported to play a role in the localization and activity of the full-length protein [18]. In particular, a 20 kDa isoform has been described as playing a critical regulatory role in the trafficking of the full-length Cx43 from the trans-Golgi network to the plasma membrane [64,65,66]. The Cx43-S3E mutations fall within the sequence of the 20 kDa isoform, which may influence its expression and, secondarily, trafficking of the full-length Cx43 to the plasma membrane. Lastly, the reduced total and membranous Cx43 in Cx43-S3E hPSC-CMs may reflect impaired protein translation, a phenomenon that has been previously reported with similar Cx43 modifications [35]. Indeed, both the mismatch between the Cx43 transcript (unchanged or even increased) and the Cx43 protein (greatly decreased) as well as the broader changes in gene expression in Cx43-S3E cultures raises the possibility of a compensatory regulatory mechanism involving microRNAs. For example, Wahl and colleagues recently reported that Cx43 expression is downregulated in mouse hPSC-CMs subjected to rapid pacing via oxidative stress and a miR-1 signaling pathway [23], and it is conceivable that the rapid spontaneous beating of Cx43-S3E could exert analogous secondary effects.

The limited expression and membranous localization of Cx43 in hPSC-CMs have been attributed to their immature state. A number of investigators have attempted to improve this and other phenotypic parameters in hPSC-CMs via pro-maturation interventions, such as prolonged duration in culture [67], culture on 2D substrates with tuned elastic modulus or nanogrooved topographies [5,68], switching of metabolic fuels from glucose to fatty acids [69], and/or co-culture with non-myocyte cell types [70]. While these strategies have usually proven successful in improving Cx43 expression, the increase is typically modest at best. Another intervention that appears to reliably influence Cx43 expression and its proper subcellular localization is the application of static or cyclic stretch [71,72,73]. In an early work, Salameh and colleagues (2010) found that cyclic mechanical stretch induced cellular elongation and increased the expression and anisotropy of Cx43 GJs in rat neonatal cardiomyocytes [73]. Similar effects on morphology and Cx43 expression have been described following the application of cyclic uniaxial stretch and/or electrical stimulation of hPSC-CM cultures, although Cx43 GJ density and subcellular localization still do not approach those of adult cardiomyocytes [74,75]. While scalability may be a challenge for therapeutic applications requiring large quantities (billions) of cells, it would be interesting to investigate in the future whether such biophysical approaches might help rescue Cx43 GJ function in hPSC-CMs either alone or when combined with Cx43 mutagenesis.

A number of electrophysiological observations in the present study also warrant some discussion. First, it is interesting that, while the Cx43-S3E hPSC-CMs exhibited greatly reduced membranous Cx43 at baseline, this effect was not accompanied by a corresponding reduction in CV by optical mapping. This outcome is not entirely surprising, given the concept of the safety factor as it has been described in adult myocardium, whereby a significant reduction in CV is observed only after substantial loss (>90%) of membranous Cx43 expression [76,77,78]. We conclude that Cx43 likely remains above this critical threshold in Cx43-S3E hPSC-CM cultures at baseline, although further reduction following ischemic challenge is clearly sufficient to impair propagation. Of course, other factors in addition to reduced GJ density contribute to slow conduction velocity in hPSC-CM cultures, including their relatively low expression of fast sodium currents and small cell size [79,80,81,82]. While we intend to directly measure sodium currents in future patch-clamp studies, we observed only a trend toward lower *SCN5A* transcripts in Cx43-S3E vs. WT hPSC-CM cultures that did not reach statistical significance.

A secondary electrophysiological observation of interest pertains to the significantly faster spontaneous beating rate in Cx43-S3E versus WT hPSC-CMs. One already mentioned potential explanation for this outcome is the compensatory changes in non-GJ-related genes, including *HCN1* and *ATP2A2*, that could plausibly accelerate voltage and/or calcium “clock” mechanisms in Cx43-S3E hPSC-CMs [83,84,85]. Moreover, even in the absence of such secondary effects on cardiac electrophysiology, mathematical modeling of electrical propagation in cardiomyocyte monolayers suggests that even modest reductions in intercellular coupling can greatly aggravate ectopy in cultures already prone to some degree of automaticity [86]. Finally, we also examined [Ca^2+^]_i_ cycling in Cx43-S3E versus WT hPSC-CMs and observed occasional irregular [Ca^2+^]_i_ transients in the former cells under spontaneous conditions. This observation suggests that Cx43-S3E hPSC-CMs may be more prone to triggered activity, at least at slower spontaneous rates of activation.

Finally, while the specific strategy pursued here to enhance graft hPSC-CM intercellular coupling and electrophysiological function via the introduction of the Cx43-S3E mutation does not appear to be a promising one, we anticipate future studies targeting other aspects of Cx43 signaling in these cells. Indeed, multiple investigators in the field have highlighted impaired Cx43 GJ-mediated intercellular communication in hPSC-CMs as a likely culprit in their predilection for pro-arrhythmic behavior [56,87,88]. While overexpressing Cx43 might seem an obvious solution, it is likely that the newly synthesized Cx43 protein would have similar challenges pertaining to transport and internalization during ischemic conditions. In addition, given the multifunctional nature of Cx43, overexpression of this target could have unintended consequences that may be deleterious to the cells in other ways. As mentioned above, other phosphorylation events are known to regulate Cx43 trafficking and GJ function [29,89]; other phosphomimetic mutations besides the Cx43-S3E mutation studied here could be tested for efficacy in hPSC-CMs, either in isolation or in combination with Cx43-S3E. Other targets in the Cx43 interactome such as Zona Occludens-1 (ZO-1) [29], N-cadherins [90], or Src kinase [91] could also be manipulated via genome engineering or pharmacological approaches.

Lastly, recent evidence suggests that Cx43 expression and function are exquisitely sensitive to oxidative stress [39,92]. Oxidative stress promotes robust cytoskeletal structure remodeling in the cell, favoring a rounded shape instead of a brick-like structure. While isolated round cardiomyocytes have been shown to survive oxidative stress, probably due to reducing damage transmission between neighboring cardiomyocytes [93], they could initiate arrhythmic behavior. Therefore, investigating strategies to improve the endogenous antioxidant response in cardiomyocytes may be a solution to reducing arrhythmias post hPSC-CM transplantation.

## 5. Conclusions

In summary, contrary to our predictions based on earlier work in transgenic mouse models [41,42], we found that gene-editing hPSC-CMs with the Cx4-S3E mutation did not enhance Cx43 expression, function, or ischemia resistance in these cells. Instead, Cx43-S3E hPSC-CMs showed substantially decreased total and membranous Cx43 at baseline that was further reduced following ischemic challenge. These outcomes were accompanied by correspondingly worsened electrophysiological behavior (increased automaticity and more irregular [Ca^2+^]_i_ cycling) and off-target changes in the expression of multiple other essential cardiomyocyte genes. These data suggest that, whether due to differences in the developmental stage or the in vitro microenvironment, the cellular mechanisms controlling Cx43 assembly and cycling likely operate quite differently in cultured hPSC-CMs versus adult ventricular myocytes in intact myocardium. Given the intense focus on hPSC-CMs as a potential cell source for cardiac regenerative medicine, more research is required to better elucidate Cx43 biology in these cells, and we anticipate that successful future efforts to improve GJ-mediated intercellular function in these cells will require interventions at multiple biological levels.

## Data Availability

The data presented in this study are available on request from the corresponding author. The data are not publicly available due to commercialization considerations.

## Supplementary Materials

The following supporting information can be downloaded at: https://www.mdpi.com/article/10.3390/biom14010061/s1, Figure S1: Cx43-S3E hPSCs karyotype. G-banded karyotype of Cx43-S3E hPSCs demonstrating a normal (46, XX) karyotype. Figure S2: Cx43-S3E hPSCs express expected markers of pluripotency. Representative confocal photomicrographs of undifferentiated Cx43-S3E hPSCs confirming strong expression of pluripotency markers NANOG (A), SOX2 (B), OCT4 (C), and SSEA4 (D). Figure S3: Full length blot detecting total Cx43 protein. Molecular ladders are depicted to the left. Figure S4: Full length blot detecting pCx43 (pS279/282) protein. Molecular ladders are depicted to the left. Table S1: Primary antibodies employed. Table S2: List of primers used. Video S1: Spontaneous beating of compact hPSC-CM monolayers. Representative videos of WT (A) and Cx43-S3E (B) hPSC-CMs at 20 days post differentiation.

## Abbreviations

hPSC	human pluripotent stem cell
hPSC-CM	human pluripotent stem cell-derived cardiomyocyte
Cx43	connexin 43
GJ	gap junction
MI	myocardial infarction
AP	action potential
APD	action potential duration
CK1	casein kinase 1
PKC	protein kinase C
MAPK	mitogen-activated protein kinase
qRT-PCR	quantitative real-time reverse transcription polymerase chain reaction
