# Supplementary material for: Casein Kinase 1 Phosphomimetic Mutations Negatively Impact Connexin-43 Gap Junctions in Human Pluripotent Stem Cell-Derived Cardiomyocytes
Source: Biomolecules. 2024 Jan 2;14(1):61. doi: 10.3390/biom14010061 (PMC10813327; doi:10.3390/biom14010061)
Supplement: Supplementary file 1 [file biomolecules-14-00061-s001.zip › biomolecules-2740493-supplementary ALL REVISION/biomolecules-2740493-supplementary REVISION.pdf]

Supplemental information

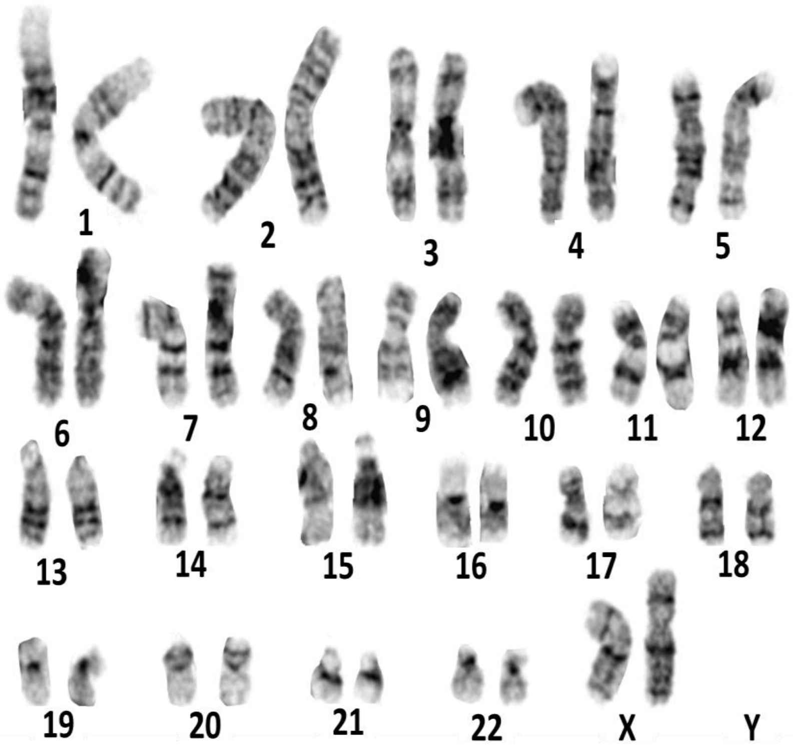

Supplemental Figure S1. Cx43-S3E hPSCs karyotype. G-banded karyotype of Cx43-S3E hPSCs demonstrating a normal (46, XX) karyotype.

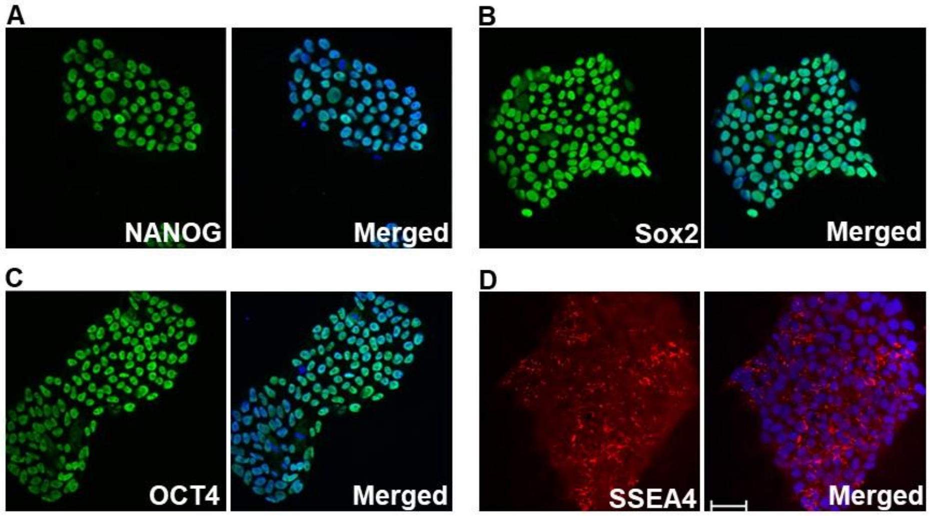

Supplemental Figure S2 Cx43-S3E hPSCs express expected markers of pluripotency. Representative confocal photomicrographs of undifferentiated Cx43-S3E hPSCs confirming strong expression of pluripotency markers NANOG (**A**), SOX2 (**B**), OCT4 (**C**), and SSEA4 (**D**).

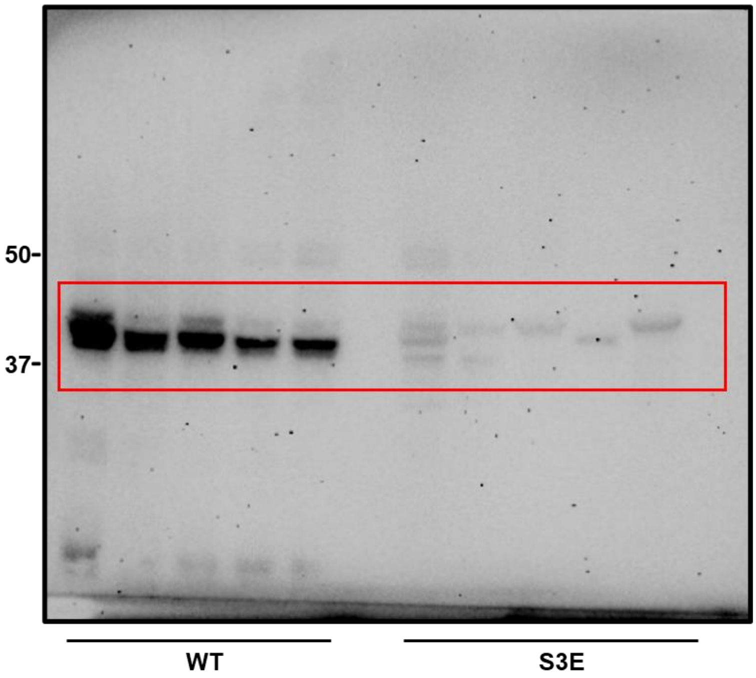

Supplemental Figure S3. Full length blot detecting total Cx43 protein. Molecular ladders are depicted to the left.

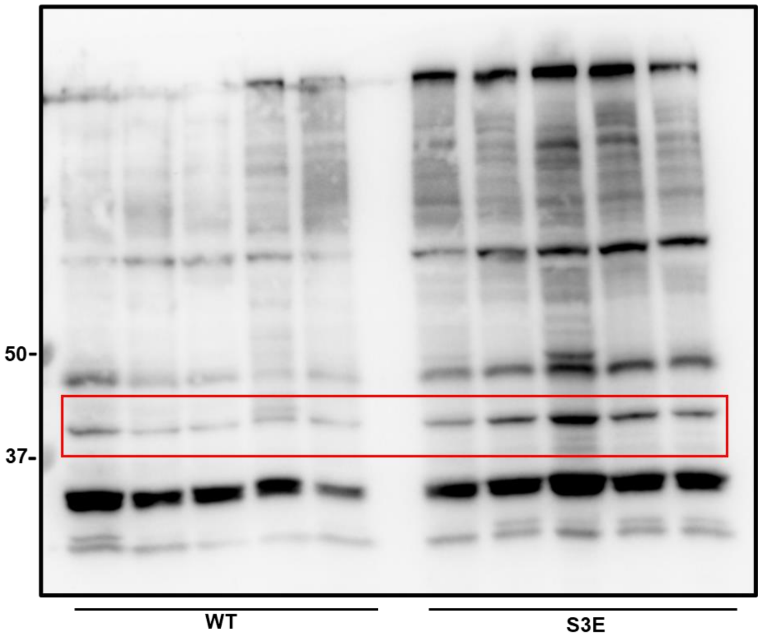

Supplemental Figure S4. Full length blot detecting pCx43 (pS279/282) protein. Molecular ladders are depicted to the left.

Supplemental Video S1. Spontaneous beating of compact hPSC-CM monolayers. Representative videos of WT **(A)** and Cx43-S3E **(B)** hPSC-CMs at 20 days post differentiation.

Supplemental Table 1: Primary antibodies employed.

**Antigen**	**Antibody type**	**Titre**	**Vendor**
**CD56**	Mouse Monoclonal (Cat#555518)	1:100	BD Biosciences

**CD140a**	Mouse Monoclonal (Cat# 556002)	1:100	BD Biosciences
**Myosin light chain-2v (MLC2v)**	Monoclonal human recombinant (clone REA401)	1:100	Miltenyi Biotec
**Cardiac troponin T (cTnT)**	Monoclonal human recombinant (clone REA400)	1:100	Miltenyi Biotec
**Connexin-43 (Cx43)**	Rabbit polyclonal (Cat # ab11370)	1:800	Abcam
**Sarcomeric α-actinin**	Mouse monoclonal (clone EA-53)	1:500	Sigma
**SSEA4**	Mouse monoclonal (Cat#4755)	1:500	Cell Signaling Technology
**Sox2**	Rabbit monoclonal (Cat#3579)	1:400	Cell Signaling Technology
**OCT4**	Rabbit monoclonal (Cat#2840)	1:200	Cell Signaling Technology
**NANOG**	Rabbit monoclonal (Cat#4903)	1:200	Cell Signaling Technology

Supplemental Table 2: List of primers used.

Target	Forward	Reverse
**Cx30**	CGTACACCAGCAGCATCTT	CGTCTGTGCTCTCTTTGATCTC
**Cx40**	AGAGTGTGAAGAAGCCCACG	AACAGATGCCAAAACTTCTGCT
**Cx40.1**	AGCGAAAAGCTGGGCAGAC	TCACACCCACTCAGACTTCCT
**Cx43**	GGTGACTGGAGCGCCTTAG	GCGCACATGAGAGATTGGGA
**Cx45**	GTCCACCCGTTTTATGTGTGC	AGTGAGTCTCGAATGGTCCCA
**Cx58**	GAAATGCCTAGGGATCGGA	AACCTAGGTGGAAAATTTCAAGA
**Cx62**	AAGGATGTCTGCTGCGTACTTA	GCCTGTTTCATCCTCAATGC
**TNNI3**	CCTCAAGCAGGTGAAGAAGG	CAGTAGGCAGGAAGGCTCAG
**TNNT2**	AGCATCTATAACTTGGAGGCAGAG	TGGAGACTTTCTGCTTATCGTTG
**MYH6**	GATAGAGAGACTCCTGCGGC	CCGTCTTCCCATTCTCGGTT
**MYH7**	TCGTGCCTGATGACAAACAGGAGT	ATACTCGGTCTCGGCAGTGACTTT
**KCNQ1**	CGCCTGAACCGAGTAGAAGA	TGAAGCATGTCGGTGATGAG
**HCN1**	CATGCCACCGCTTTAATCCAG	ATTGTAGCCACCAGTTTCCGA
**HNC2**	AGAAGGGCATTGACTCCGAG	TAGCGGATCAGGCGTGAGA
**HCN4**	TCTTCCTCATTGTGGAGACACGCA	TGAGGATCTTCGTGAAGCGGACAA
**KCNH2**	CAACCTGGGCGACCAGATAG	GGTGTTGGGAGAGACGTTGC
**KCNJ8**	GTGATTGCCGTCCGAAATGG	AGTTGGTGAATAGGAACCACCT
**KCNJ2**	CTTTGTAGTGCCAGAGACTTAG	CACTGTCGTCTTCCTCTTTG
**KCND2**	CTACCTGTTCCGGTGATTGTATCC	TCTTTTGTGCCCTTCGTTTGT
**SCN5A**	TCTCTATGGCAATCCACCCCA	GAGGACATACAAGGCGTTGGT
**ATP2A2**	CATCAAGCACACTGATCCCGT	CCACTCCCATAGCTTTCCCAG
**CACNA1C**	TGATTCCAACGCCACCAATTC	GAGGAGTCCATAGGCGATTACT
**CAMKIIB**	GCACACCAGGCTACCTGTC	CATACGCCTCTTTGCGAAGG
**PLN**	ACCTCACTCGCTCAGCTATAA	CATCACGATGATACAGATCAGCA
**TRDN**	TCACAGAAGACATAGTGACGACG	TGGCAATAGAGCTTGCTGAAA
**RYR2**	CATCGAACACTCCTCTACGGA	GGACACGCTAACTAAGATGAGGT
**JPH2**	ACTCTGGCTCCTGGAACTTTG	GCGCCCCTTGGTCTCTATG
**DDB1**	TCAACGGCATGATAGGGCTG	CGCTCGGTGTGAAAGGATCT
