# Supplementary figures and images for: Casein Kinase 1 Phosphomimetic Mutations Negatively Impact Connexin-43 Gap Junctions in Human Pluripotent Stem Cell-Derived Cardiomyocytes
Source: Biomolecules. 2024 Jan 2;14(1):61. doi: 10.3390/biom14010061 (PMC10813327; doi:10.3390/biom14010061)

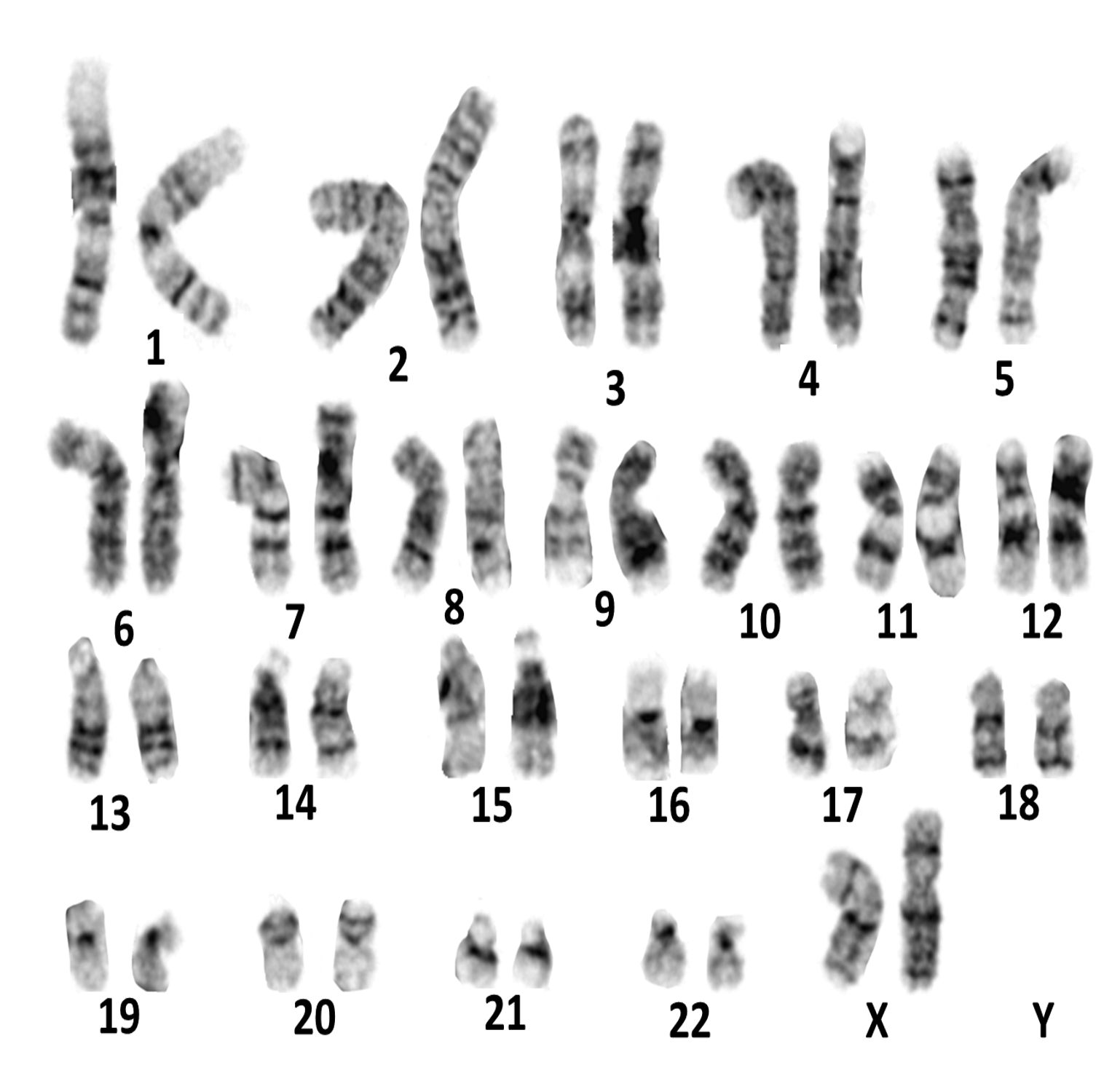

Supplement: Supplementary file 1 [file biomolecules-14-00061-s001.zip › biomolecules-2740493-supplementary ALL REVISION/Figure S1.tif]

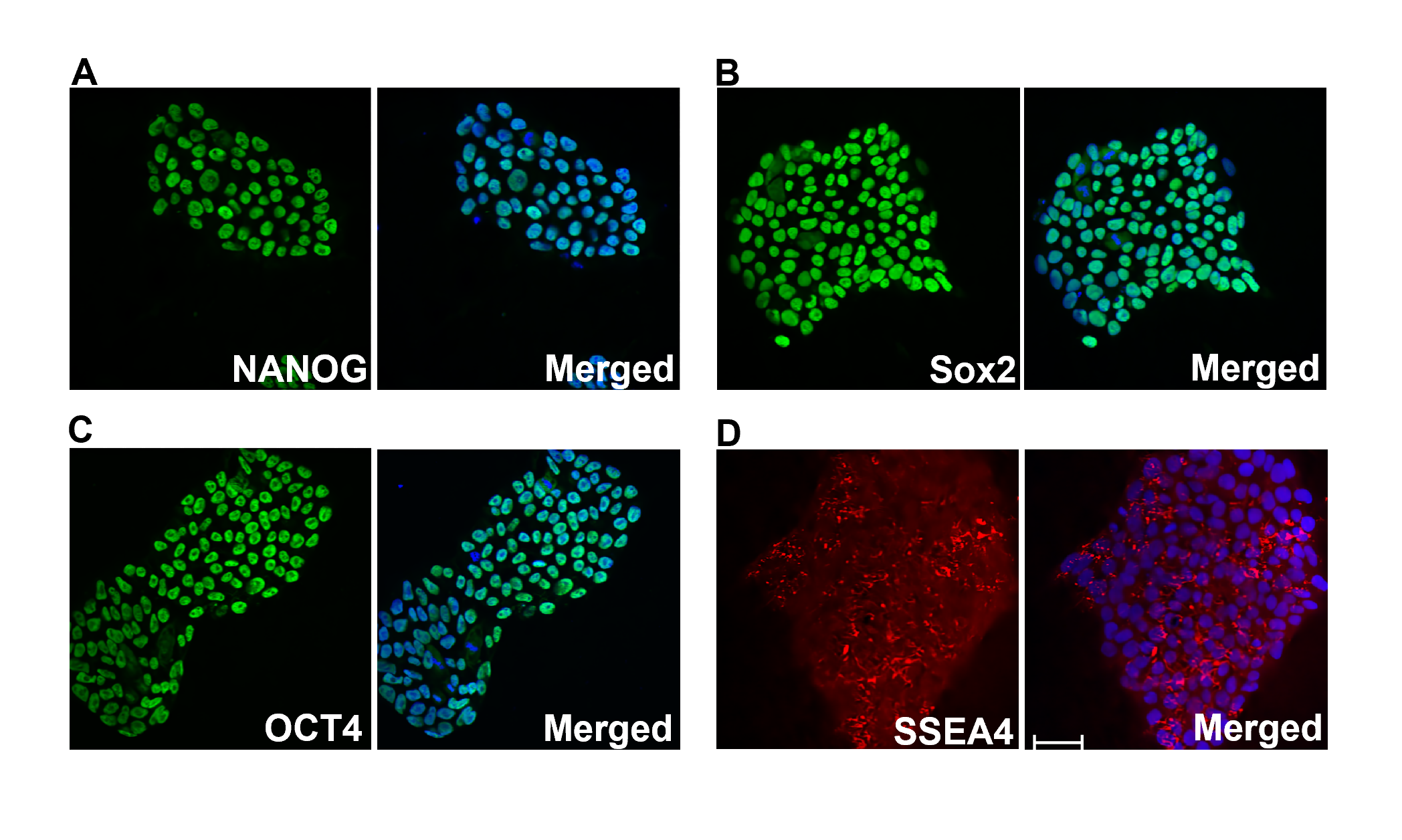

Supplement: Supplementary file 1 [file biomolecules-14-00061-s001.zip › biomolecules-2740493-supplementary ALL REVISION/Figure S2.tif]

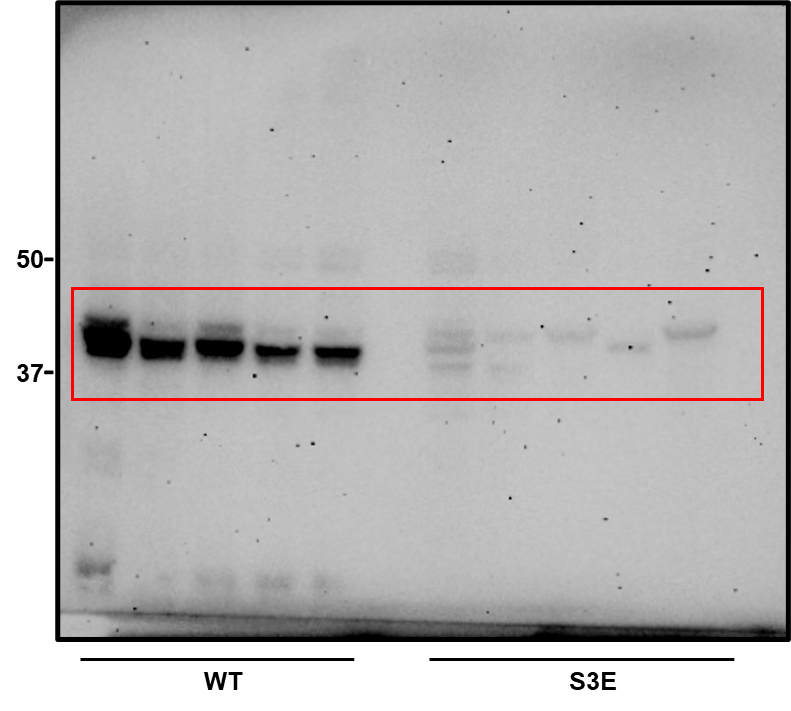

Supplement: Supplementary file 1 [file biomolecules-14-00061-s001.zip › biomolecules-2740493-supplementary ALL REVISION/Figure S3.tif]

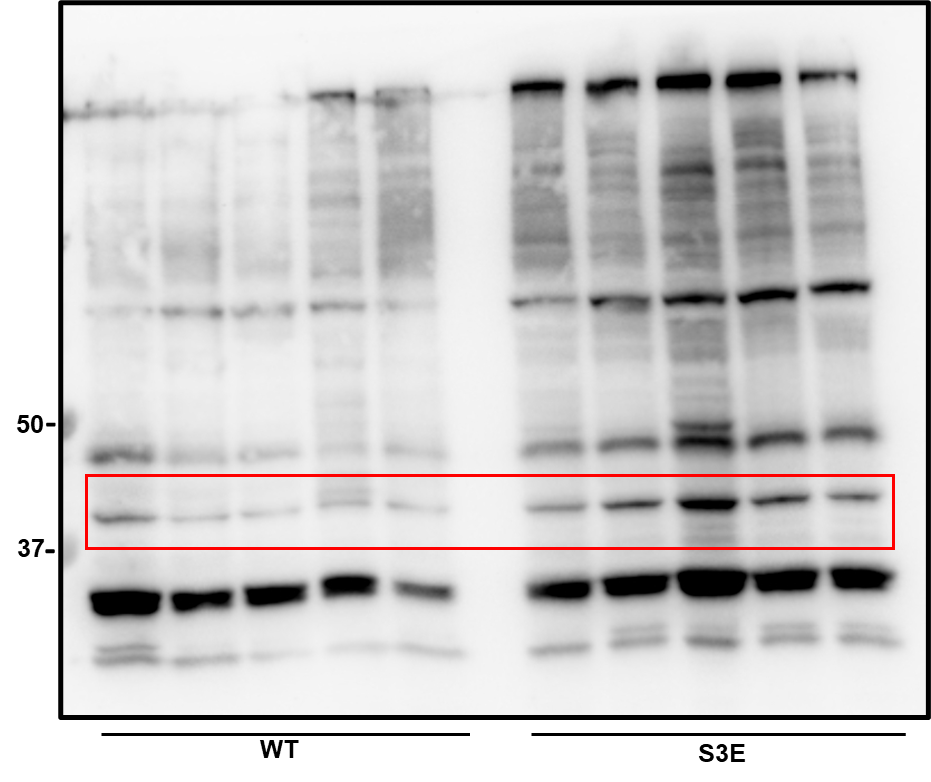

Supplement: Supplementary file 1 [file biomolecules-14-00061-s001.zip › biomolecules-2740493-supplementary ALL REVISION/Figure S4.tif]
